# Socio-economic factors associated with the incidence of Shiga-toxin producing *Escherichia coli* (STEC) enteritis and cryptosporidiosis in the Republic of Ireland, 2008–2017

**DOI:** 10.1017/S0950268821001564

**Published:** 2021-07-29

**Authors:** E. Cleary, M. Boudou, C. ÓhAiseadha, P. McKeown, P. Garvey, J. O'Dwyer, P. Hynds

**Affiliations:** 1 SpatioTemporal Environmental Epidemiology Research (STEER) Group, Environmental Sustainability & Health Institute, Technological University Dublin, Dublin, Ireland; 2 Health Service Executive, (HSE), Dublin, Ireland; 3 Health Protection Surveillance Centre (HPSC), Dublin, Ireland; 4 School of Biological, Earth and Environmental Sciences, University College Cork, Cork, Ireland; 5 Water and Environment Research Group, Environmental Research Institute, Cork, Ireland; 6 Irish Centre for Research in Applied Geoscience, University College Dublin, Dublin, Ireland

**Keywords:** Cryptosporidiosis, VTEC, STEC, ecological study, socioeconomic drivers, Ireland

## Abstract

The Republic of Ireland (ROI) currently reports the highest incidence rates of Shiga-toxin producing *Escherichia coli* (STEC) enteritis and cryptosporidiosis in Europe, with the spatial distribution of both infections exhibiting a clear urban/rural divide. To date, no investigation of the role of socio-demographic profile on the incidence of either infection in the ROI has been undertaken. The current study employed bivariate analyses and Random Forest classification to identify associations between individual components of a national deprivation index and spatially aggregated cases of STEC enteritis and cryptosporidiosis. Classification accuracies ranged from 78.2% (STEC, urban) to 90.6% (cryptosporidiosis, rural). STEC incidence was (negatively) associated with a mean number of persons per room and percentage of local authority housing in both urban and rural areas, addition to lower levels of education in rural areas, while lower unemployment rates were associated with both infections, irrespective of settlement type. Lower levels of third-level education were associated with cryptosporidiosis in rural areas only. This study highlights settlement-specific disparities with respect to education, unemployment and household composition, associated with the incidence of enteric infection. Study findings may be employed for improved risk communication and surveillance to safeguard public health across socio-demographic profiles.

## Introduction

Shiga-toxin producing *Escherichia coli* (STEC) is an enteric pathogen causing gastroenteritis with symptoms ranging from diarrhoea and haemorrhagic colitis to haemolytic uraemic syndrome, and death in severe cases [1, 2]. *Cryptosporidium* spp. are protozoan parasites characterised by self-limiting diarrhoeal disease in immunocompetent people, but with potentially severe clinical symptoms among immunocompromised individuals, young children and older adults [3, 4]. Over the past decade, crude incidence rates (CIRs) of STEC enteritis and cryptosporidiosis in Ireland have been among the highest in Europe, with CIRs of 20.0 (STEC) and 13.2 (cryptosporidiosis) cases per 100 000 population, respectively, notified during 2018 [5, 6].

The spatial distribution of both infections exhibits a strong urban–rural partition in Ireland, with the incidence rates of both infections significantly higher in rural areas [7–9]. Dominant transmission pathways include direct contact with farm animals, consumption from private (ground)water sources and high reliance on (poorly managed) private domestic wastewater treatment systems (i.e. septic tanks); all factors typically associated with rurality [8–10]. International studies have reported associations between infection incidence and a broad range of social determinants, including measured deprivation [11–13]. For example, the incidence of food- and waterborne infections in urban areas is frequently associated with poor sanitation infrastructure, synonymous with economically deprived areas and person-to-person transmission (i.e. elevated population densities) [11–13]. Conversely, infectious diseases in rural areas have been attributed to barriers accessing healthcare [14] and poor water resource infrastructure [15].

While the environmental determinants of STEC enteritis and cryptosporidiosis in Ireland are relatively well understood (e.g. private well use, high vulnerability hydrogeological settings, high livestock densities, wastewater infrastructure) [8, 15], to date, the socio-economic drivers of infection have not been comprehensively explored. Accordingly, the current study sought to examine the presence of associations between individual components of the Pobal HP deprivation index [16] with spatially aggregated cases of STEC enteritis and cryptosporidiosis in Ireland from 2008 to 2017, with analyses delineated by urban/rural classification due to the aforementioned divide associated with both infections in Ireland.

## Methods

### Infection data

Laboratory-confirmed, irriversibly anonymised case data were obtained from the Computerised Infectious Disease Reporting (CIDR) database (http://www.hpsc.ie/CIDR/), an information system used for collating notifiable (communicable) infection data in Ireland [17]. Primary and index cases of STEC enteritis were included for analyses, with primary and index cases defined as sporadic (i.e. not associated with a confirmed outbreak or cluster) or the first case identified as part of a recognised outbreak/cluster, respectively [8]. Confirmed STEC cases occurring between 1 January 2013 and 31 December 2017 were included for analyses, with STEC cases notified prior to 2013 excluded to ensure consistency regarding laboratory confirmation protocols.

Data for notified primary and secondary cases of cryptosporidiosis were obtained from 1 January 2008 to 31 December 2017, with a secondary infection defined as a laboratory-confirmed case with an epidemiological link to another notified case [18]. Cryptosporidiosis cases prior to 1 January 2008 were excluded to avoid biases associated with a large cryptosporidiosis outbreak which occurred in the west of Ireland during spring 2007 [19]. All individual cases of infection were geo-referenced to one of 18 488 Central Statistics Office (CSO) Small Area (SA) centroids, using a previously developed protocol [20]. SAs comprise 80–120 dwellings, geographically defined for population census reporting, and are the smallest spatially referenced unit in Ireland [21]. A binary variable was developed to indicate the presence/absence of a confirmed infection within all individual SAs over the study period.

### Pobal HP deprivation index data

**Fig. 1.**
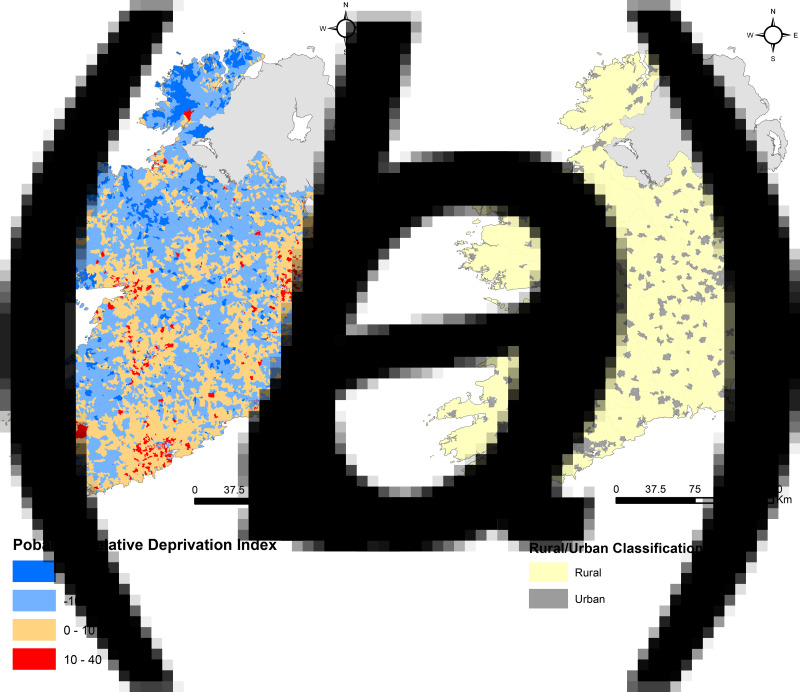
(a) Pobal HP relative deprivation index spatial distribution by CSO small area in Ireland. (b) Urban/rural classification spatial distribution by CSO small area in Ireland.

The Pobal Haase Pratschke (HP) deprivation index comprises 16 individual components representing the three main dimensions of deprivation, as measured in the Republic of Ireland (ROI); demographic profile, social class composition and labour market situation (Table 1; Fig. 1) [16]. Absolute and relative index scores represent composite measures of deprivation based on these components, calculated for each SA and measured on a single scale across all census periods (Supplementary Table S1). The absolute deprivation score reflects any changes to the national economy at SA level between census periods while the relative deprivation index score is a comparative measure of deprivation between SAs during a census period [16]. Deprivation index component data were obtained for two waves of the national census of Ireland (2011 and 2016) to correspond with the study period.

**Table 1.** Pobal HP deprivation index components and descriptions

Component label	Component name	Component description
HPabs	Absolute HP index score	Composite measure of deprivation calculated for each SA, measured on a single scale across all census periods
HPrel	Relative HP index score	A measure of the level of deprivation in each SA relative to all other small areas surveyed
TOTPOP	Total population	Total population in each SA during each census period
POPCHG	Population change	Percentage increase in population over the previous five years
AGEDEP	Age dependency rate	Percentage of population aged under 15 or over 64 years of age
LONEPA	Lone parent ratio	Percentage of households with children aged under 15 years and headed by a single parent
EDLOW	Primary education	Percentage of people in each SA with primary education as their highest level of education attainment
EDHIGH	Third-level education	Percentage of people in each SA with third-level education as their highest level of education attainment
HLPROF	Higher and lower professionals	Percentage of households headed by professionals or managerial and technical employees, including farmers with 100 acres or more
LSKILL	Proportion of semi-skilled and unskilled manual workers	Percentage of households in each SA headed by semi-skilled or unskilled manual workers, including farmers with less than 30 acres
UNEMPM	Male unemployment rate	Rate of male unemployment in each SA
UNEMPF	Female unemployment rate	Rate of female unemployment in each SA
PEROOM	Persons per room	Mean number of persons per household room in each small area
LARENT	Local authority housing	Percentage of local authority housing in each SA
PRRENT	Privately rented housing	Percentage of privately rented housing in each SA
OHOUSE	Own home	Percentage of privately owned housing in each SA

*Note.* Reprinted from the 2016 Pobal HP Deprivation Index for Small Areas (SA): Introduction and Reference Tables, Trutz Haase Jonathan Pratschke, September 2017.

### Urban/rural classification

A binary SA-specific settlement type variable was developed using data obtained from the Irish CSO (Fig. 1b; Supplementary Table S2). The CSO settlement type dataset comprises 14 variables classified along an urban/peri-urban/rural scale ranging from ‘city’ (1) to ‘rural’ (14). The urban/rural variable was coded such that any classification which included a built-up area (classification 1–13) was recoded as ‘urban’, with remaining areas (classification 14) coded as ‘rural’. All datasets were spatially integrated using a unique SA identifier in Stata V14 (StataCorp, College Station, Texas, USA).

### Data analysis

#### Bivariate analysis

Exploratory analyses of associations between individual deprivation components and the incidence of both infections in rural and urban areas were conducted via sequential bivariate logistic regressions. Statistically significant associations were assessed using a confidence level of 95% (*P* ⩽ 0.05), with explanatory variables for multivariate classification selected based on Bayesian Inference Criterion (BIC). The presence of collinearity between explanatory variables was diagnosed using the Variance Inflation Factor (VIF) and Spearman's rank (non-parametric) correlation coefficient (*ρ*). A VIF of ⩾5 and a Spearman's *ρ* of ⩾0.5 were considered indicative of potentially significant collinearity between individual deprivation components.

#### Random Forest classification

A suite of classification algorithms (support vector machines, Naïve Bayes, optimised generalised linear models and CART (decision trees)) were tested, with the Random Forest (RF) algorithm found to exhibit the highest classification performance. Calibrated parameters were selected using hyperparameter tuning and validated via an optimised iterative Breiman's RF parameter estimation algorithm in the randomForest package (R Foundation for Statistical Computing, Vienna, Austria). Parameters for STEC and cryptosporidiosis models, obtained from hyperparameter tuning, were set at a number of randomly sampled variables at each split (mtry = 3–4). RF models were calibrated using partitioned training datasets (70% of data) and ‘re-trained’ using full datasets (100% of data) to ensure classification stability (i.e. change in variable importance between datasets). Attribute importance was assessed via the mean Gini value decrease, with larger values indicative of a greater contribution to infection classification.

## Results

### Bivariate analyses

#### STEC enteritis

Overall, 2757 confirmed cases of STEC enteritis were geocoded to one of 12 246 categorically urban SAs and 6242 rural SAs; 2342 SAs were identified as having ⩾1 case of STEC enteritis, of which 1088 were urban (46.5% of total STEC SA level infection; 5.9% of total SAs; 8.9% of total urban SAs; OR = 0.31); and 1254 were rural (53.5% of total STEC SA level infection; 6.8% of total SAs; 20% of total rural SAs; OR = 3.25). A significantly higher proportion of STEC cases occurred among children ⩽5 years (39.6% of total infections) and people aged ⩾65 years (16.6% of total infections). Infection was relatively evenly distributed among males (47.3%) and females (52.7%).

The occurrence of STEC enteritis was significantly associated with both absolute (presence mean = −4.69; absence mean = −5.35; *P* *=* 0.002) and relative deprivation scores (presence mean = −0.90; absence mean = −1.54; *P* *=* 0.002) in rural areas only (Tables 2, 3 and S3). STEC infection was also associated with a higher SA total population in both urban (282.6 *vs.* 264.4; *P* < 0.001) and rural areas (260.5 *vs.* 236.1; *P* = 0.001), and a significantly higher age dependency rate (AGEDEP) in urban areas (34.3 *vs.* 32.3; *P* < 0.001). The occurrence of STEC enteritis exhibited negative associations with household density (PERROOM) in urban areas (0.53 *vs.* 0.55; *P* < 0.001) and percentage of local authority housing (LARENT) in both urban (8.9% *vs.* 10.9%; *P* < 0.001) and rural SAs (3.5% *vs.* 4.3%; *P* = 0.001). Within rural SAs, STEC presence was associated with a lower incidence of third-level education (29.1% *vs.* 29.7%; *P* < 0.001), and a lower incidence of secondary-level education (i.e. primary education only) in both urban and rural areas (18.2% *vs.* 15.5%; *P* = 0.001). STEC presence was associated with lower mean unemployment rates among both male (11.4% *vs.* 12.9%; *P* = 0.001) and female residents (9.7% *vs.* 10.4%; *P* = 0.001) across both settlement types.

**Table 2.** Calculated odds ratios (ORs) for STEC (presence/absence) and individual Pobal HP deprivation index components in urban areas across both census periods

	STEC Urban 2011				STEC Urban 2016			
Variable	Cl 2.5%	OR	Cl 97.5%	Sig (*P*)	Cl 2.5%	OR	Cl 97.5%	Sig (*P*)
HPabs	0.9975	1.0033	1.0091	0.2620	0.9983	1.0039	1.0095	0.1752
HPrel	0.9975	1.0030	1.0085	0.2892	0.9974	1.0029	1.0085	0.3032
AGEDEP	1.0100	1.0171	1.0243	<0.0001	1.0138	1.0212	1.0287	<0.0001
EDHIGH	0.9967	1.0000	1.0033	0.9908	0.9968	1.0000	1.0032	0.9880
EDLOW	0.9876	0.9932	0.9987	0.0165	0.9844	0.9909	0.9973	0.0061
HLPROF	1.0018	1.0053	1.0089	0.0033	1.0020	1.0055	1.0090	0.0023
LARENT	0.9876	0.9920	0.9962	0.0003	0.9873	0.9916	0.9958	0.0001
LONEPA	0.9878	0.9916	0.9953	<0.0001	0.9862	0.9903	0.9944	<0.0001
LSKILL	0.9854	0.9912	0.9969	0.0029	0.9848	0.9906	0.9964	0.0016
OHOUSE	1.0049	1.0075	1.0102	0.0001	1.0049	1.0075	1.0102	<0.0001
PEROOM	0.2265	0.3780	0.6096	0.0001	0.2574	0.4109	0.6382	0.0001
POPCHG	0.9919	1.0013	1.0075	0.7311	0.7981	1.0785	1.4111	0.6028
PRRENT	0.9912	0.9943	0.9972	0.0002	0.9912	0.9943	0.9974	0.0003
TOTPOP	1.0010	1.0017	1.0023	<0.0001	1.0011	1.0016	1.0022	<0.0001
UNEMPF	0.9863	0.9926	0.9987	0.0192	0.9823	0.9890	0.9956	0.0013
UNEMPM	0.9884	0.9930	0.9976	0.0031	0.9851	0.9908	0.9963	0.0014

**Table 3.** Calculated odds ratios (ORs) for STEC (presence/absence) and individual Pobal HP deprivation index components in rural areas across both census periods

	STEC Rural 2011				STEC Rural 2016			
Variable	Cl 2.5%	OR	Cl 97.5%	Sig (*P*)	Cl 2.5%	OR	Cl 97.5%	Sig (*P*)
HPabs	0.9955	1.0060	1.0167	0.2630	1.0058	1.0152	1.0248	0.0016
HPrel	0.9960	1.0060	1.0161	0.2389	1.0051	1.0146	1.0241	0.0024
AGEDEP	0.9911	1.0044	1.0179	0.5190	0.9933	1.0066	1.0201	0.3319
EDHIGH	0.9825	0.9897	0.9969	0.0055	0.9864	0.9932	0.9999	0.0488
EDLOW	0.9805	0.9884	0.9963	0.0042	0.9781	0.9869	0.9958	0.0041
HLPROF	0.9968	1.0031	1.0094	0.3424	1.0014	1.0076	1.0138	0.0169
LARENT	0.9764	0.9859	0.9951	0.0034	0.9744	0.9839	0.9930	0.0008
LONEPA	0.9847	0.9907	0.9967	0.0026	0.9805	0.9869	0.9931	<0.0001
LSKILL	0.9796	0.9885	0.9974	0.0119	0.9845	0.9935	1.0025	0.1610
OHOUSE	1.0108	1.0169	1.0233	<0.0001	1.0094	1.0154	1.0216	<0.0001
PEROOM	0.3260	0.8330	1.3275	0.6164	0.4359	1.0875	2.5968	0.8536
POPCHG	0.9659	0.9981	1.0146	0.8538	0.8295	1.4546	2.5219	0.1859
PRRENT	0.9658	0.9754	0.9847	<0.0001	0.9705	0.9800	0.9892	<0.0001
TOTPOP	1.0029	1.0037	1.0045	<0.0001	1.0027	1.0035	1.0042	<0.0001
UNEMPF	0.9716	0.9803	0.9888	<0.0001	0.9746	0.9842	0.9937	0.0013
UNEMPM	0.9819	0.9885	0.9950	0.0006	0.9647	0.9734	0.9819	<0.0001

#### Cryptosporidiosis

**Fig. 2.**
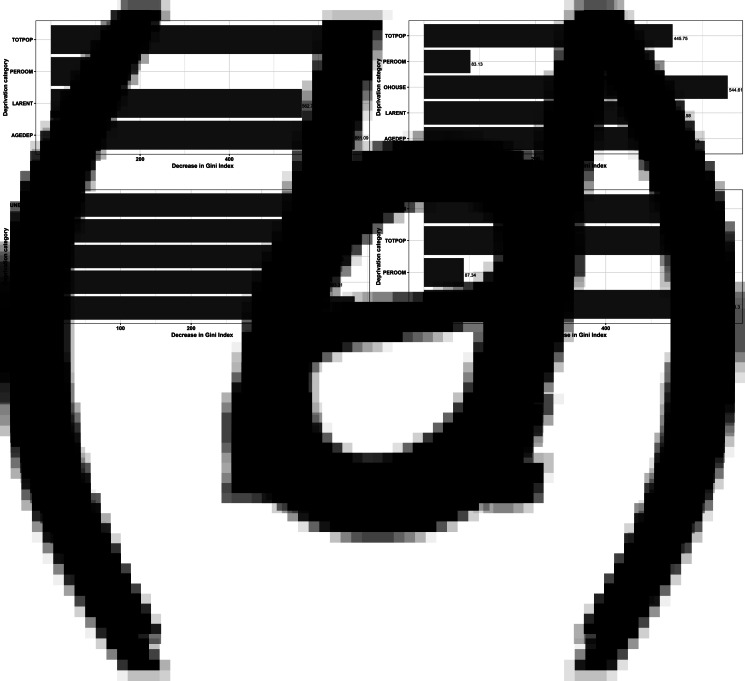
Mean decrease in Gini index for retained explanatory Pobal HP index deprivation components in Random Forest models using 2011 and 2016 census deprivation examined at both urban and rural level; (a) Cryptosporidiosis Urban 2011; (b) Cryptosporidiosis Urban 2016; (c) Cryptosporidiosis Rural 2011; (d) Cryptosporidiosis Rural 2016.

Overall, 4509 confirmed cases of cryptosporidiosis were geocoded to 3412 SAs, of which 2027 SAs were rural (59.4% of infections; 11.0% of total SAs; 32.5% of rural SAs; OR = 3.77) and 1385 were urban (40.6% of infections; 7.5% of total SAs; 11.3% of urban SAs; OR = 0.27) (Fig. 2). Just under 60% of infections were observed among children aged ⩽5 years, with no significant difference noted with respect to case gender (male = 53%, female = 47%).

As for STEC enteritis, the incidence of cryptosporidiosis was associated with both higher absolute (presence mean = −4.9; absence mean = −5.4; *P* = 0.006) and relative deprivation scores (presence mean = −1.1; absence mean = −1.5; *P* = 0.006) in rural areas only (Tables 4, 5 and S4). Cryptosporidiosis occurrence across both settlement types was associated with a higher total population (e.g. 2011 Urban 271.1 *vs.* 251.1; *P* < 0.001) and age dependency rate (e.g. 2011 Urban 33.3 *vs.* 31.9; *P* < 0.001) (Tables 5, S3 and S6).

**Table 4.** Calculated odds ratios (ORs) for cryptosporidiosis (presence/absence) and individual Pobal HP deprivation index components in urban areas across both census periods

	Crypto Urban 2011				Crypto Urban 2016			
Variable	Cl 2.5%	OR	Cl 97.5%	Sig (*P*)	Cl 2.5%	OR	Cl 97.5%	Sig (*P*)
HPabs	0.9931	0.9983	1.0035	0.5189	0.9906	0.9955	1.0005	0.0782
HPrel	0.9933	0.9982	1.0032	0.4829	0.9898	0.9947	0.9997	0.0358
AGEDEP	1.0191	1.0256	1.0322	<0.0001	1.0219	1.0287	1.0355	<0.0001
EDHIGH	0.9946	0.9976	1.0006	0.1115	0.9929	0.9958	0.9987	0.0047
EDLOW	0.9882	0.9932	0.9981	0.0075	0.9894	0.9951	1.0008	0.0922
HLPROF	0.9988	1.0021	1.0053	0.2089	0.9977	1.0008	1.0040	0.6098
LARENT	0.9949	0.9984	1.0018	0.3623	0.9947	0.9982	1.0016	0.3059
LONEPA	0.9860	0.9895	0.9928	<0.0001	0.9883	0.9919	0.9955	<0.0001
LSKILL	0.9929	0.9980	1.0031	0.4472	0.9945	0.9996	1.0046	0.8670
OHOUE	1.0058	1.0082	1.0106	<0.0001	1.0051	1.0075	1.0099	<0.0001
PEROM	0.1254	0.2061	0.3320	<0.0001	0.1372	0.2163	0.3355	<0.0001
POPCHG	0.9852	0.9974	1.0048	0.5806	0.7209	0.9595	1.2447	0.7666
PRRENT	0.9869	0.9898	0.9926	<0.0001	0.9875	0.9904	0.9933	<0.0001
TOTPOP	1.0017	1.0023	1.0029	<0.0001	1.0014	1.0019	1.0024	<0.0001
UNEMF	0.9926	0.9981	1.0034	0.4816	0.9968	1.0025	1.0080	0.3878
UNEMM	0.9949	0.9990	1.0030	0.6148	0.9945	0.9994	1.0041	0.7977

**Table 5.** Calculated odds ratios (ORs) for cryptosporidiosis (presence/absence) and individual Pobal HP deprivation index components in rural areas across both census periods

	Crypto Rural 2011				Crypto Rural 2016			
Variable	Cl 2.5%	OR	Cl 97.5%	Sig (*P*)	Cl 2.5%	OR	Cl 97.5%	Sig (*P*)
HPabs	0.99622	1.00524	1.0144	0.256	1.00319	1.01124	1.0194	0.006183
HPrel	0.99688	1.0054	1.014	0.2148	1.00215	1.01014	1.0182	0.01296
AGEDEP	1.01148	1.02315	1.035	<0.0001	1.00735	1.0189	1.0306	0.001291
EDHIGH	0.98585	0.99198	0.9981	0.1115	0.98981	0.99556	1.0013	0.1317
EDLOW	0.99275	0.9994	1.0061	0.8603	0.98807	0.99554	1.003	0.2428
HLPROF	0.99754	1.00292	1.0083	0.2879	1.012	1.0013	1.00662	0.01475
LARENT	0.97795	0.98577	0.9934	0.0003351	0.97612	0.98387	0.9914	<0.0001
LONEPA	0.98261	0.98775	0.9928	<0.0001	0.97786	0.98326	0.9886	<0.0001
LSKILL	0.98047	0.98804	0.9956	0.1115	0.98165	0.98937	0.9971	0.007226
OHOUSE	1.010091	1.015089	1.0202	<0.0001	1.010543	1.015537	1.0207	<0.0001
PEROOM	0.24941	0.59966	1.1203	0.1115	0.51102	1.109	2.3666	0.7907
POPCHG	0.98638	1.00336	1.0201	0.6526	0.72899	1.18583	1.9208	0.4898
PRRENT	0.97325	0.9809	0.9884	<0.0001	0.97241	0.98023	0.9879	<0.0001
TOTPOP	1.00233	1.00303	1.0037	<0.0001	1.00211	1.00276	1.0034	<0.0001
UNEMPF	0.97257	0.97986	0.9871	<0.0001	0.97634	0.98443	0.9925	0.0001748
UNEMPM	0.98271	0.98827	0.9938	<0.0001	0.97298	0.98007	0.9871	<0.0001

The occurrence of cryptosporidiosis exhibited a negative association with household density (0.52 *vs.* 0.55; *P* < 0.001) in urban areas only, while a lower lone parent ratio (12.8 *vs.* 14.5; *P* < 0.001) and lower proportion of semi-skilled or unskilled workers (17.7 *vs.* 18.2; *P* = 0.008) were associated with infection irrespective of settlement type. Cryptosporidiosis was associated with lower proportions of residents with primary education only (13.6 *vs.* 14.5; *P* = 0.008) in urban areas only, whereas incidence across all settlement types was associated with a lower proportion of residents with third-level education (36.9% *vs.* 38.5%). Lower male (11.8 *vs.* 12.9; *P* < 0.001) and female unemployment rates (9.8 *vs.* 10.5; *P* < 0.001) were correlated with cryptosporidiosis in rural areas only.

### Random Forest classification

#### STEC enteritis

**Fig. 3.**
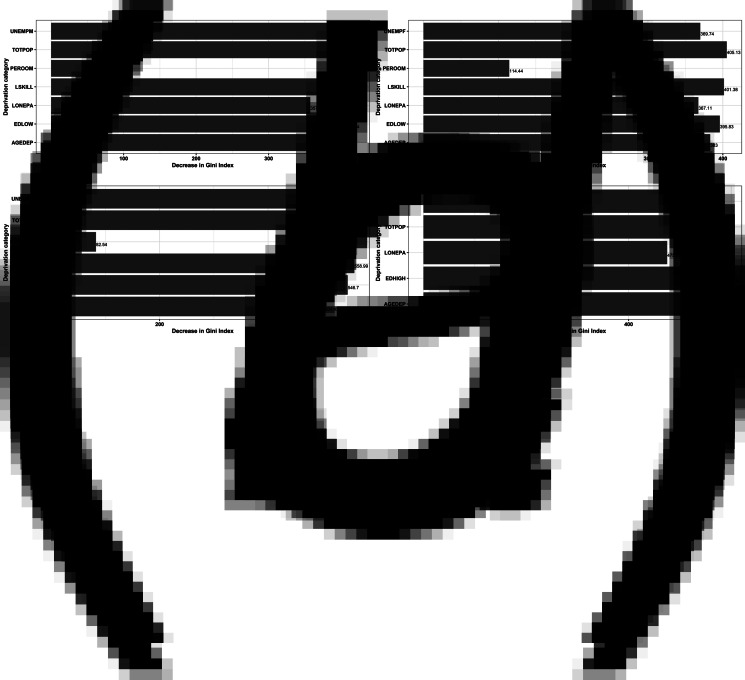
Mean decrease in Gini index for retained explanatory Pobal HP index deprivation components in Random Forest models using 2011 and 2016 census deprivation examined at both urban and rural level: (a) STEC Urban 2011, (b) STEC Urban 2016, (c) STEC Rural 2011, (d) STEC Rural 2016.

Classification accuracy for STEC presence/absence ranged from 78.2% (Urban 2016) to 85.2% (Rural 2011) (after 100% model re-training) (Table S5). Total population (TOTPOP) exhibited a high relative contribution to infection classification within all models (highest mean Gini decrease [MGD] = 622.93, Urban 2011) (Fig. 3). A lower mean number of persons per room was associated with the presence of STEC enteritis within both urban and rural SAs, albeit this was the lowest relative contributor in all three models in which it appeared (Urban 2011, 2016; Rural 2016).

In urban SAs, higher age dependency rates (highest MDG = 681.1, Urban 2011) and lower percentages of local authority housing (highest MDG = 562.7, Urban 2011) were significant contributors to STEC classification, with neither of these attributes retained in rural models. Conversely, a lower proportion of population with third-level education (highest MDG = 668.3, Rural 2016) and primary level education only (highest MDG = 393.3, Rural 2011) were associated with the occurrence of STEC enteritis in rural areas only. Similarly, rates of (un)employment were explanatory within rural areas only; lower rates of female unemployment were associated with the occurrence of STEC enteritis using 2011 deprivation index data (highest MDG = 396.1, Rural 2011), while lower rates of male unemployment were associated with infection during the 2016 census period (highest MDG = 604.6; Rural 2016).

#### Cryptosporidiosis

Classification accuracy for cryptosporidiosis ranged from 82.0% (Urban 2016) to 90.6% (Rural 2011) (Table S6). Higher total SA populations (highest MDG = 574.9, Rural 2016) and age dependency rates (highest MDG = 550.9, Rural 2016) were significant attributes within all four classification models (Fig. 2). A lower household density was also significant within both rural and urban areas, although this attribute exhibited the lowest overall relative contribution to infection classification (highest MDG = 114.4, Urban 2016).

Across both settlement types, rates of unemployment were significantly associated with the presence of cryptosporidiosis, with associations again alternating based on gender-specific rates and time period; female unemployment was significant within the 2016 Rural (MDG = 519.5) and Urban RF models (MDG = 369.7), while conversely, male unemployment was significant within both rural models (2011 – MDG = 405.6; 2016 – MDG = 529.5). A lower population proportion with primary education only was retained within urban cryptosporidiosis models (highest MDG = 406.2, Urban 2011), while a lower proportion with third-level education were associated with infection within rural areas only (highest MDG = 591.4, Rural 2016). A higher proportion of skilled and semi-skilled workers were associated with cryptosporidiosis in urban settlements only (highest MDG = 418.2, Urban 2011).

## Discussion

The present study represents the first comprehensive investigation of individual socio-economic index components, as they relate to the incidence of two enteric infections in the ROI. Prior to the interpretation of study findings, the authors feel it is important to highlight some inherent limitations of the current study. As with any ecological analyses employing spatial areas as the primary study unit, it is crucial that caution is employed when interpreting study findings or replicating this approach. The Pobal HP deprivation index was designed and is employed as a measure of national-scale socio-economic deprivation, and as such, was not specifically developed for elucidating the transmission of or exposure to enteric pathogens and/or public health risk. Moreover, as both deprivation indices and infection data were aggregated to spatial units, examination of individual-level associations of infection with socio-economic elements is not possible, nor should study findings be interpolated to this level, i.e. findings are susceptible to ecological fallacy whereby affluent individuals (and cases of infection) may reside within a socio-economically deprived area, and *vice versa*. Comparison of findings for the two infections should account for differential management of secondary cases; while secondary cases were excluded from the STEC dataset, all cluster-related cases were included in the cryptosporidiosis dataset. This approach was taken partially to minimise spatiotemporal bias, and partially to account for differences in transmission. A significant majority of STEC clusters were consistently identified as such in the CIDR database across all geographical areas and throughout the data collection period, thus it was possible to consistently exclude secondary cases. Conversely, cryptosporidiosis clusters were not all identified in the earlier years of the 10-year dataset, and so secondary cases could not be excluded without introducing a spatiotemporal bias. Additionally, the route of transmission for STEC clusters was almost exclusively person-to-person, thus secondary cases contribute little to the statistical power of presented analyses. In contrast, the suspected route of transmission for cryptosporidiosis outbreaks was typically waterborne or animal contact, thus statistical source attribution is justified. The use of binary classification (i.e. RF) as opposed to a zero-inflated count (e.g. Poisson) model should minimise any potential effect this differential management of secondary infections had on presented findings.

Bivariate analyses reveal some similarities between several index components and the incidence of both STEC enteritis and cryptosporidiosis, which is likely reflective of inherent similarities between the two pathogens (e.g. sources, transmission pathways, etc.). Higher total population number was associated with both organisms in both urban and rural areas and across both census time periods. Higher local populations (and thus density) are often associated with economic deprivation, particularly in urban areas; previous studies have shown that the transmission of diarrhoeal diseases is typically higher among populations in more socially deprived urban areas [22]. Moreover, settlement patterns in Ireland are characterised by a burgeoning rural ‘commuter belt’, whereby rural populations exhibit a preference for living in the suburban periphery of large rural towns or urban connurbations due to high urban house prices and/or proximity to workplaces and schools [8]. Accordingly, ‘peri-urban’ populations, while characteristically rural with respect to local landuse and infrastructure, may be more susceptible to infection via secondary (person-to-person) transmission through increased contact with urban residents via childcare facilities and occupational exposures, in addition to being reliant on characteristically ‘rural’ infrastructure (e.g. private wells, septic tanks).

A higher age dependency rate was also associated with the occurrence of both infections. In Ireland, children under 5 years of age represent a high-risk group for STEC infection, with cryptosporidiosis also a leading cause of paediatric gastrointestinal morbidity [23]. The association between infection incidence and age dependency rate mirrors the demographic breakdown of cases, with young children (<5 years of age) and older adults (>65 years of age) historically characterised by the highest rates of infection [9, 17]. Young children may be at increased risk due to frequent contact with other young children, lower standards of hygiene and inherent immunological susceptibility to infection [24], with onward transmission of infection to older members of households also attributed to higher rates of infection among children [25].

The incidence of STEC enteritis in rural areas was significantly associated with lower levels of third-level education, likely reflecting the prevalence of agriculturally based employment in rural Ireland, which is typically generational and not contingent on higher education. Notably, significant associations were also identified between the presence of both infections and lower rates of male and female unemployment in rural areas only. Lower levels of poverty, in concurrence with higher mean income, have been associated with STEC incidence in the USA and Japan [26, 27], although this association has not been found across all regions (e.g. Finland [28], Canada [29]). The interplay between affluence and infection (notification) may be indicative of healthcare-seeking behaviours among higher income sub-populations as suggested by Lal *et al*. [30] who report that increasingly deprived areas were inversely associated with the incidence of cryptosporidiosis in New Zealand. Further support is provided by a previous study of waterborne cryptosporidiosis in Ireland which reported that paediatric cases from agricultural backgrounds were less likely to have unemployed parents than a control group (children with gastroenteritis not associated with *Cryptosporidium*) [31]. Conversely, higher rates of female and male unemployment were associated with the presence of cryptosporidiosis in urban areas, suggesting that the relationship between affluence and cryptosporidiosis is not nationally uniform.

Lower mean household density was associated with the incidence of both infections in urban areas; this finding was unexpected based on previous studies which report household size and structural profile as a proxy for secondary transmission [32, 33]. Household transmission and overcrowding are important risk factors for cryptosporidiosis, albeit more frequently reported within low- and middle-income countries [34]. The association between enteric infections and urban household density identified in the current study may indicate elevated infection risks among affluent demographic groups. Likewise, a lower proportion of local authority housing was associated with STEC presence in urban areas, once again potentially reflecting the nexus between pathogenic exposure and economic affluence. Previous studies have cited international travel and food consumption patterns associated with higher socio-demographic status (e.g. higher intake of fresh fruit, vegetables and unprocessed produce) as risk factors for both STEC enteritis and cryptosporidiosis [27, 28].

Education represents a key indicator of socio-economic status, and by extension, overall population health, with increased educational attainment typically associated with a lower incidence of non-communicable disease [35, 36]. The current study identified significant associations between infection and areas characterised by higher proportions of (a) primary education only and (b) a third-level education; thus, higher and lower educational attainment were significantly associated with the incidence of both infections at the local level. A recent review by Newman *et al*. [37] reports significant, non-ordered variation between educational attainment and the incidence of STEC enteritis. In Ireland, younger cohorts are more likely to have received a third-level education than their predecessors, due to the introduction of free secondary school education in the late-1960s, thus the proportion of people with primary education only is appreciably higher among older people [38], as such, lower educational attainment may represent a potential proxy measure for immunological status. Conversely, previous work reports that higher infection risks are associated with higher levels of education [39], with higher education serving as a proxy for increased affluence, which as previously mentioned is often associated with international travel and consumption of unprocessed foods [27, 28].

Overall, infection classification based on RF models using deprivation index components was generally considered moderately accurate; based on the published literature, this (unsurprisingly) suggests that the incidence (and thus mechanics) of infection within spatial units cannot be predicted solely based upon socio-economic metrics, with environmental factors, for example, likely to significantly strengthen predictive capacity, particularly in rural areas. Environmental drivers of STEC enteritis and cryptosporidiosis typically exhibit marked differences with respect to settlement type, for example, the incidence of STEC enteritis in rural areas has been associated with direct (farm) animal contact and contaminated drinking water, while contaminated food products have been reported as a significant driver in urban areas [10, 39, 40, 41]. Notwithstanding, full (i.e. 100% datasets) RF models presented here did exhibit 50–80% ‘true-positive’ classification (i.e. sensitivity), thus there is little doubt that both infections are driven, to some extent, by local socio-economic profile in Ireland. Accordingly, the authors recommend that future work focus on increasingly prospective approaches (in parallel with retrospective, data-driven studies), and employ a variety of metrics/indices for the classification of socio-economic profile. Moreover, the authors strongly recommend that future research should seek to incorporate socio-economic, environmental (including climactic) and behavioural drivers of infection to provide increasingly holistic models of infection incorporating the myriad sources, pathways and receptors potentially associated with transmission.

## Conclusion

Increased local age dependency rates and total population number were associated with the occurrence of both infections, irrespective of settlement type, highlighting population density (person-to-person contact) and vulnerable sub-populations (i.e. immunological status, infection severity) as important transmission drivers. Numerous heterogeneities were identified with respect to ‘place’ as it relates to both settlement type and socio-economic profile, and particularly those associated with education, unemployment and household composition. Lower rates of third-level education, unemployment and household density were associated with infections in urban areas only. Differences were also observed between socio-economic components and infection aetiology; lower proportions of lone parent households and higher proportions of semi-skilled and unskilled workers were associated with the incidence of cryptosporidiosis, but not STEC enteritis. Future work should incorporate environmental (including climactic), socio-economic and epidemiological data to increase current understanding of spatially-specific exposure to and transmission of enteric infections, thus permitting improved evidence-based surveillance, targeted intervention design and mitigation strategies, both nationally and internationally.

## Data Availability

The human infection data that support the findings of this study are available from the Computerised Infectious Disease Reporting (CIDR) Committee (https://www.hpsc.ie/cidr/), and require formal permission following application by researchers for individual studies. Restrictions apply to the availability of these data, which were used under licence for this study. The urban/rural classification used to support the findings of this study is freely available from the Central Statistics Office (Ireland) (https://www.cso.ie/en/releasesandpublications/ep/p-cp3oy/cp3/urr/). The Pobal HP deprivation index data used to support the findings of this study are freely available, and may be accessed from http://trutzhaase.eu/deprivation-index/the-2016-pobal-hp-deprivation-index-for-small-areas/.

## Supplementary material

For supplementary material accompanying this paper visit http://dx.doi.org/10.1017/S0950268821001564. click here to view supplementary material
