# Supplementary material for: Socio-economic factors associated with the incidence of Shiga-toxin producing Escherichia coli (STEC) enteritis and cryptosporidiosis in the Republic of Ireland, 2008–2017
Source: Epidemiol Infect. 2021 Jul 29;149:e184. doi: 10.1017/S0950268821001564 (PMC8365853; doi:10.1017/S0950268821001564)
Supplement: Supplementary file 1 [file S0950268821001564sup001.docx]

**Supplementary materials**

| **Table S1. Pobal HP Index absolute and relative deprivation index score classification** | |
| --- | --- |
| **Index Score** | **Level of deprivation/affluence** |
| **-40 to - 30** | Extremely disadvantaged |
| **-30 to -20** | Very disadvantaged |
| **-20 to -10** | Disadvantaged |
| **-10 to 0** | Marginally below average |
| **0 to 10** | Marginally above average |
| **10 to 20** | Affluent |
| **20 to 30** | Very affluent |
| **30 to 40** | Extremely affluent |

*Note.* Reprinted from the 2016 Pobal HP Deprivation Index for Small Areas

(SA): Introduction and Reference Tables, Trutz Haase Jonathan Pratschke,

September 2017

| **Table S2. Central Statistics Office urban/rural classification in the Republic of Ireland** |
| --- |

| **SA classification** | | **Urban/rural classification** |
| --- | --- | --- |
| 01 | = | Cities |
| 02 | = | City suburbs |
| 03 | = | Mixed city suburbs and towns with a population of 5,000 or higher |
| 04 | = | Mixed city suburbs and towns with a population of 1,000 - 4,999 |
| 05 | = | Mixed city suburbs, towns with a population of 5,000 or higher and rural |
| 06 | = | Mixed city suburbs, towns with a population of 1,000 - 4,999 and rural |
| 07 | = | Mixed city suburbs and rural |
| 08 | = | Towns with a population of 5,000 or higher |
| 10 | = | Mixed towns with a population of 5,000 or higher and rural |
| 11 | = | Mixed towns with a population of 5,000 or higher, towns with a population of 1,000 - 4,999 and rural |
| 12 | = | Towns with a population of 1,000 - 4,999 |
| 13 | = | Mixed towns with a population of 1,000 - 4,999 and rural |
| 14 | = | Rural |

| **Table S3. Pobal HP Deprivation Index component mean differences between STEC enteritis presence/absence delineated by urban/rural classification in the Republic of Ireland, 2013-2017** | | | | | | | | |
| --- | --- | --- | --- | --- | --- | --- | --- | --- |
|  | **STEC Urban 2011** | | **STEC Urban 2016** | | **STEC Rural 2011** | | **STEC Rural 2016** | |
| **Variable** | **STEC presence** | **STEC absence** | **STEC presence** | **STEC absence** | **STEC presence** | **STEC absence** | **STEC presence** | **STEC absence** |
| **HPabs** | -5.36 | -5.73 | -3.19 | -3.66 | -8.18 | -8.39 | -4.69 | -5.35 |
| **HPrel** | 1.25 | .89 | 1.04 | .68 | -1.62 | -1.85 | -0.90 | -1.54 |
| **TOTPOP** | 266.64 | 252.10 | 282.65 | 264.36 | 256.15 | 233.34 | 260.53 | 236.12 |
| **POPCHG** | 0.71 | 0.63 | 0.06 | 0.05 | 0.17 | 0.19 | 0.01 | 0.01 |
| **AGEDEP** | 32.35 | 31.00 | 34.32 | 32.75 | 35.91 | 35.82 | 37.64 | 37.50 |
| **LONEPA** | 23.19 | 25.63 | 21.43 | 23.85 | 12.96 | 13.98 | 12.89 | 14.21 |
| **EDLOW** | 13.58 | 14.44 | 11.52 | 12.39 | 18.57 | 19.29 | 15.53 | 16.18 |
| **EDHIGH** | 33.66 | 33.69 | 38.33 | 38.33 | 24.18 | 24.94 | 29.14 | 29.72 |
| **HLPROF** | 35.69 | 34.07 | 37.12 | 35.43 | 34.17 | 33.87 | 36.33 | 35.58 |
| **LSKILL** | 17.92 | 18.95 | 17.50 | 18.59 | 17.65 | 18.21 | 17.81 | 18.12 |
| **UNEMPM** | 22.71 | 24.00 | 14.45 | 15.63 | 20.78 | 21.83 | 11.39 | 12.87 |
| **UNEMPF** | 15.86 | 16.61 | 12.58 | 13.59 | 12.57 | 13.62 | 9.75 | 10.43 |
| **PEROOM** | 0.51 | 0.53 | 0.53 | 0.55 | 0.47 | 0.47 | 0.47 | 0.47 |
| **LARENT** | 7.86 | 9.78 | 8.90 | 10.96 | 3.31 | 3.99 | 3.49 | 4.28 |
| **PRRENT** | 23.73 | 26.28 | 23.61 | 26.00 | 6.91 | 8.12 | 7.72 | 8.68 |
| **OHOUSE** | 67.08 | 62.71 | 66.05 | 61.70 | 87.32 | 85.35 | 86.26 | 84.43 |

| **Table S4. Pobal HP Deprivation Index component mean differences between cryptosporidiosis presence/absence delineated by urban/rural classification in the Republic of Ireland, 2013-2017** | | | | | | | | |
| --- | --- | --- | --- | --- | --- | --- | --- | --- |
|  | **Crypto Urban 2011** | | **Crypto Urban 2016** | | **Crypto Rural 2011** | | **Crypto Rural 2016** | |
| **Variable** | **Crypto presence** | **Crypto absence** | **Crypto presence** | **Crypto absence** | **Crypto presence** | **Crypto absence** | **Crypto presence** | **Crypto absence** |
| **HPabs** | -5.87 | -5.68 | -4.12 | -3.56 | -8.22 | -8.40 | -4.89 | -5.38 |
| **HPrel** | 0.72 | 0.95 | 0.12 | 0.79 | -1.67 | -1.88 | -1.11 | -1.55 |
| **TOTPOP** | 271.19 | 251.13 | 284.78 | 263.59 | 250.22 | 232.00 | 253.7 | 234.93 |
| **POPCHG** | 0.53 | 0.65 | 0.05 | 0.06 | .22 | .18 | .01 | .01 |
| **AGEDEP** | 32.92 | 30.89 | 34.78 | 32.65 | 36.17 | 35.68 | 37.80 | 37.40 |
| **LONEPA** | 22.71 | 25.76 | 21.83 | 23.87 | 12.86 | 14.22 | 12.81 | 14.49 |
| **EDLOW** | 13.59 | 14.47 | 11.89 | 12.37 | 19.12 | 19.15 | 15.90 | 16.12 |
| **EDHIGH** | 32.93 | 33.78 | 36.93 | 38.51 | 24.39 | 24.98 | 29.35 | 29.7 |
| **HLPROF** | 34.77 | 34.15 | 35.81 | 35.55 | 34.12 | 33.84 | 36.17 | 35.52 |
| **LSKILL** | 18.64 | 18.88 | 18.45 | 18.50 | 17.71 | 18.29 | 17.72 | 18.22 |
| **UNEMPM** | 23.70 | 23.91 | 15.45 | 15.54 | 20.89 | 21.97 | 11.79 | 12.95 |
| **UNEMPF** | 16.36 | 16.57 | 13.71 | 13.47 | 12.68 | 13.76 | 9.84 | 10.51 |
| **PEROOM** | 0.51 | 0.54 | 0.52 | 0.55 | 0.46 | 0.47 | 0.47 | 0.47 |
| **LARENT** | 9.23 | 9.66 | 10.34 | 10.83 | 3.37 | 4.08 | 3.56 | 4.39 |
| **PRRENT** | 22.17 | 26.55 | 22.27 | 26.24 | 7.19 | 8.21 | 7.82 | 8.80 |
| **OHOUSE** | 67.31 | 62.56 | 65.99 | 61.59 | 87.00 | 85.14 | 86.09 | 84.18 |

| **Table S5. Confusion matrix (classification accuracy) for STEC enteritis presence/absence using Random Forest classification, delineated by settlement type (urban/rural) and census period (2011, 2016)** | | | | |
| --- | --- | --- | --- | --- |
| **STEC enteritis urban SA 2011 Pobal HP deprivation data** | | | | |
| Classification on full dataset using model fit on training dataset | | | Correctly classified (%) | |
|  | Predicted STEC presence | Predicted STEC absence |  |  |
| Observed STEC presence | 554 | 534 | STEC presence | 50.9 |
| Observed STEC absence | 99 | 11,059 | STEC absence | 99.1 |
| Mean prediction accuracy on full dataset: 94.8% | | |  |  |
|  | | |  | |
| Classification on full dataset using model fit on full dataset | | | Correctly classified (%) | |
|  | Predicted STEC presence | Predicted STEC absence |  |  |
| Observed STEC presence | 857 | 231 | STEC presence | 78.8 |
| Observed STEC absence | 29 | 11,129 | STEC absence | 99.7 |
| Mean prediction accuracy on full dataset: 97.9% | | |  |  |
|  | | | | |
| **STEC enteritis urban SA 2016 Pobal HP deprivation data** | | |  |  |
| Classification on full dataset using model fit on training dataset | | | Correctly classified (%) | |
|  | Predicted STEC presence | Predicted STEC absence |  |  |
| Observed STEC presence | 567 | 518 | STEC presence | 52.2 |
| Observed STEC absence | 61 | 11,080 | STEC absence | 99.4 |
| Mean prediction accuracy on full dataset: 95.3% | | |  |  |
|  | | |  | |
| Classification on full dataset using model fit on full dataset | | | Correctly classified (%) | |
|  | Predicted STEC presence | Predicted STEC absence |  |  |
| Observed STEC presence | 849 | 236 | STEC presence | 78.2 |
| Observed STEC absence | 12 | 11,129 | STEC absence | 99.7 |
| Mean prediction accuracy on full dataset: 97.9% | | |  |  |
|  | | | | |
| **STEC enteritis rural SA 2011 Pobal HP deprivation data** | | | | |
| Classification on full dataset using model fit on training dataset | | | Correctly classified (%) | |
|  | Predicted STEC presence | Predicted STEC absence |  |  |
| Observed STEC presence | 818 | 436 | STEC presence | 65.2 |
| Observed STEC absence | 276 | 4712 | STEC absence | 94.5 |
| Mean prediction accuracy on full dataset: 88.6 | | |  |  |
|  | | |  | |
| Classification on full dataset using model fit on full dataset | | | Correctly classified (%) | |
|  | Predicted STEC presence | Predicted STEC absence |  |  |
| Observed STEC presence | 1068 | 186 | STEC presence | 85.2 |
| Observed STEC absence | 1540 | 4948 | STEC absence | 76.4 |
| Mean prediction accuracy on full dataset: 96.4% | | |  |  |
|  | | | | |
| **STEC enteritis rural SA 2016 Pobal HP deprivation data** | | | | |
| Classification on full dataset using model fit on training dataset | | | Correctly classified (%) | |
|  | Predicted STEC presence | Predicted STEC absence |  |  |
| Observed STEC presence | 821 | 433 | STEC presence | 65.4 |
| Observed STEC absence | 209 | 4776 | STEC absence | 95.8 |
| Mean prediction accuracy on full dataset: 89.7% | | |  |  |
|  | | |  | |
| Classification on full dataset using model fit on full dataset | | | Correctly classified (%) | |
|  | Predicted STEC presence | Predicted STEC absence |  |  |
| Observed STEC presence | 1060 | 194 | STEC presence | 84.5 |
| Observed STEC absence | 54 | 4931 | STEC absence | 98.9 |
| Mean prediction accuracy on full dataset: 96.0% | | |  |  |

| **Table S6. Confusion matrix (classification accuracy) for cryptosporidiosis presence/absence using Random Forest algorithm, delineated by settlement type (urban/rural) and census period (2011, 2016)** | | | | | | | |
| --- | --- | --- | --- | --- | --- | --- | --- |
| **Cryptosporidiosis urban SA 2011 Pobal HP deprivation data** | | | | | | | |
| Classification on full dataset using model fit on training dataset | | | | | |  | |
|  | | Predicted crypto presence | | Predicted crypto absence | | Correctly classified (%) | |
| Observed crypto. presence | | 711 | | 673 | | Crypto. presence | 51.4 |
| Observed crypto. absence | | 126 | | 10,688 | | Crypto. absence | 98.8 |
| Mean prediction accuracy on full dataset: 93.6 | | | | | |  |  |
|  | | | | | |  | |
| Classification on full dataset using model fit on full dataset | | | | | |  | |
|  |  | Predicted crypto presence | | Predicted crypto absence | | Correctly classified (%) | |
| Observed crypto. presence | | 1137 | | 247 | | Crypto. presence | 82.1 |
| Observed crypto. absence | | 21 | | 10,793 | | Crypto. absence | 99.8 |
| Mean prediction accuracy on full dataset: 97.8% | | | | | |  |  |
|  |  |  | |  | |  |  |
| **Cryptosporidiosis urban SA 2016 Pobal HP deprivation data** | | | | | | | |
| Classification on full dataset using model fit on training dataset | | | | | |  | |
|  |  | Predicted crypto presence | | Predicted crypto absence | | Correctly classified (%) | |
| Observed crypto. presence | | 714 | | 668 | | Crypto. presence | 51.7 |
| Observed crypto. absence | | 117 | | 10,685 | | Crypto. absence | 98.9 |
| Mean prediction accuracy on full dataset: 93.4% | | | | | |  |  |
|  | | | | | |  | |
| Classification on full dataset using model fit on full dataset | | | | | |  | |
|  |  | Predicted crypto presence | | Predicted crypto absence | | Correctly classified (%) | |
| Observed crypto. presence | | 1134 | | 248 | | Crypto. presence | 82.0 |
| Observed crypto. absence | | 25 | | 10,777 | | Crypto. absence | 99.8 |
| Mean prediction accuracy on full dataset: 97.8% | | | | | |  |  |
|  | | | | | | | |
| **Cryptosporidiosis rural SA 2011 Pobal HP deprivation data** | | | | | | | |
| Classification on full dataset using model fit on training dataset | | | | | |  | |
|  | | Predicted crypto presence | | Predicted crypto absence | | Correctly classified (%) | |
| Observed crypto. presence | | 1500 | | 527 | | Crypto. presence | 74.0 |
| Observed crypto. absence | | 364 | | 3851 | | Crypto. absence | 91.4 |
| Mean prediction accuracy on full dataset: 85.7% | | | | | |  |  |
|  | | | | | |  | |
| Classification on full dataset using model fit on full dataset | | | | | |  | |
|  |  | Predicted crypto presence | | Predicted crypto absence | | Correctly classified (%) | |
| Observed crypto. presence | | 1837 | | 190 | | Crypto. presence | 90.6 |
| Observed crypto. absence | | 71 | | 4144 | | Crypto. absence | 98.3 |
| Mean prediction accuracy on full dataset: 95.8% | | | | | |  |  |
|  |  |  | |  | |  |  |
| **Cryptosporidiosis rural SA 2016 Pobal HP deprivation data** | | | | | | | |
| Classification on full dataset using model fit on training dataset | | | | | |  | |
|  | | | Predicted crypto presence | | Predicted crypto absence | Correctly classified (%) | |
| Observed crypto. presence | | 1497 | | 529 | | Crypto. presence | 73.9 |
| Observed crypto. absence | | 366 | | 3846 | | Crypto. absence | 91.3 |
| Mean prediction accuracy on full dataset: 85.6% | | | | | |  |  |
|  | | | | | |  | |
| Classification on full dataset using model fit on full dataset | | | | | |  | |
|  |  | Predicted crypto presence | | Predicted crypto absence | | Correctly classified (%) | |
| Observed crypto. presence | | 1833 | | 193 | | Crypto. presence | 90.5 |
| Observed crypto. absence | | 77 | | 4135 | | Crypto. absence | 98.2 |
| Mean prediction accuracy on full dataset: 95.7% | | | | | |  |  |
